# Quantitative Analysis of Mechanisms That Govern Red Blood Cell Age Structure and Dynamics during Anaemia

**DOI:** 10.1371/journal.pcbi.1000416

**Published:** 2009-06-26

**Authors:** Nicholas J. Savill, William Chadwick, Sarah E. Reece

**Affiliations:** 1 Centre for Infectious Diseases, Institute of Immunology and Infection Research, University of Edinburgh, Edinburgh, United Kingdom; 2 Institute of Evolutionary Biology, University of Edinburgh, Edinburgh, United Kingdom; Lilly Singapore Centre for Drug Discovery, Singapore

## Abstract

Mathematical modelling has proven an important tool in elucidating and quantifying mechanisms that govern the age structure and population dynamics of red blood cells (RBCs). Here we synthesise ideas from previous experimental data and the mathematical modelling literature with new data in order to test hypotheses and generate new predictions about these mechanisms. The result is a set of competing hypotheses about three intrinsic mechanisms: the feedback from circulating RBC concentration to production rate of immature RBCs (reticulocytes) in bone marrow, the release of reticulocytes from bone marrow into the circulation, and their subsequent ageing and clearance. In addition we examine two mechanisms specific to our experimental system: the effect of phenylhydrazine (PHZ) and blood sampling on RBC dynamics. We performed a set of experiments to quantify the dynamics of reticulocyte proportion, RBC concentration, and erythropoietin concentration in PHZ-induced anaemic mice. By quantifying experimental error we are able to fit and assess each hypothesis against our data and recover parameter estimates using Markov chain Monte Carlo based Bayesian inference. We find that, under normal conditions, about 3% of reticulocytes are released early from bone marrow and upon maturation all cells are released immediately. In the circulation, RBCs undergo random clearance but have a maximum lifespan of about 50 days. Under anaemic conditions reticulocyte production rate is linearly correlated with the difference between normal and anaemic RBC concentrations, and their release rate is exponentially correlated with the same. PHZ appears to age rather than kill RBCs, and younger RBCs are affected more than older RBCs. Blood sampling caused short aperiodic spikes in the proportion of reticulocytes which appear to have a different developmental pathway than normal reticulocytes. We also provide evidence of large diurnal oscillations in serum erythropoietin levels during anaemia.

## Author Summary

Red blood cells are made in the bone marrow and released into the blood stream. Their population is kept at equilibrium by complex negative feedback mechanisms. Genetic diseases, pathogens such as malaria, and extreme environmental changes can alter this equilibrium, either directly by prematurely killing red blood cells or indirectly through disrupting these feedback mechanisms. In this paper we test competing hypotheses about the nature of some of these mechanisms by fitting mathematical models to experimental data. Our results give us a better understanding of the mechanisms that govern the red blood cell population and will improve models of diseases that affect red blood cells.

## Introduction

Mathematical modelling has proven an important tool in elucidating and quantifying the mechanisms that govern physiological processes. Here we are concerned with understanding the processes responsible for the age structure and population dynamics of red blood cells (RBC). There are two main fields of research developing mathematical models of the dynamics of the erythropoietic system. Interestingly, both fields appear to progress in isolation from each other. The first, pioneered by Mackey [1], has mainly focused on explaining periodic haematological diseases by exploring how the feedback from the circulating RBC population influences self-renewal, maturation and apoptosis of RBC progenitors (erythroid progenitors) in bone marrow ([2]–[9], see [10] for a detailed review of the literature). The approach is fairly theoretical: modifications to previous models—in the light of new evidence in the literature—are examined from a dynamical systems point of view, often with an emphasis on integrating partial differential equation models to time-delay ordinary differential equation models. Model testing against real data is rarely undertaken ([9] is one exception), and fits are qualitative. The second field of research, pioneered by Veng-Pedersen and co-workers [11], has focused on developing pharmaceutic PK/PD models to explain perturbations to RBC, immature RBC (reticulocyte), erythropoietin (Epo) and haemoglobin dynamics induced by phlebotomy, recombinant human Epo or chemotherapeutic drugs [12]–[18]. This approach is directed at explaining reticulocyte dynamics as this is commonly used for clinical diagnoses and management of anaemia (see [16] and references therein). Model fitting to data and parameter estimation is integral to this approach.

One of our main areas of study is early blood stage malaria infection of mammalian hosts, particularly in mice as these are our main experimental model of malaria infection. Malaria parasites invade RBCs, killing them in the process and causing, in some cases, severe anaemia. Some species also prefer to invade reticulocytes over normocytes [19]. In our work, we use mathematical models to elucidate and quantify the processes that govern RBC and malaria parasite dynamics in infections [20]. The standard model for RBC production in malaria infection models is to assume that RBC growth rate is proportional to the instantaneous difference in normal and anaemic RBC concentrations (see, for example, [21],[22]). Extensions to this model include a time lag between RBC concentration and growth rate [20] and age structure [23]. Although relatively simple, these models may be sufficient to explain malaria infection data. Whether they are or not, however, has never been adequately tested.

The aim of the work described in this paper is to gain a better understanding of the processes that govern age structure and dynamics of reticulocyte and RBC concentrations under anaemic conditions in general. The results can then be used to inform our mathematical models of malaria infections. A complicating factor with modelling the erythropoietic system in malaria infections is that the immune responses against the parasite are known to interact with the erythropoietic system [24]. It is therefore easier to elucidate erythropoietic mechanisms under anaemic conditions in the absence of immune responses. One method of inducing anaemia without an immune response is by administering phenylhydrazine (PHZ). PHZ has been used for over 100 years as a means to induce haemolytic anaemias in experimental animals [25]. For our purposes it perturbs the RBC population away from equilibrium. The resulting transient dynamics give us a window into the mechanisms that govern RBC population dynamics.

Quantitative fitting of mathematical models to data integrated with statistical analysis is a powerful and sensitive method of testing and comparing hypotheses and for development of evidence based models. Here, we attempt to fit a set of mathematical models constructed from combinations of sets of competing hypotheses to some new experimental data from our lab. These hypotheses reflect uncertainty in the qualitative and quantitative nature of mechanisms that govern RBC age structure and dynamics.

In particular, there are five mechanisms we wish to examine in detail: the feedback from RBC concentration to production rate of reticulocytes in bone marrow, the release of reticulocytes from bone marrow and their subsequent ageing and clearance in the circulation, the effect of PHZ on RBC age structure, and the effect of blood sampling on reticulocyte proportion.

The preliminary phase of the work reported here was a trial model development and fitting exercise to some existing unpublished data on PHZ treated mice. These data consisted of RBC and reticulocyte concentrations measured on day 0 and days 3–8 after PHZ treatment. Reticulocytes take about 1 to 2 days to mature into normocytes in the circulation. In normal conditions they are present at very low concentrations of about 1% of total RBCs. Anaemia causes their proportion to rise dramatically as production of erythroid progenitors in the bone marrow is up-regulated in response to increased Epo production in the kidney. The reticulocyte proportion is an excellent variable to test hypotheses about erythropoietic mechanisms because cells pass rapidly through this development stage, thus providing a much finer scale resolution on RBC dynamics than total RBC concentration.

The trial model fitting exercise informed the experiments described in this paper. We used more mice (25 instead of 3) to improve statistical inference. We sampled for longer (114 days instead of 9) to examine return to equilibrium. We sampled twice per day on days 5 to 9 to characterise the fast reticulocyte dynamics that occur during this period. We measured serum Epo concentrations to relate RBC concentration during anaemia to production and release rates of bone marrow reticulocytes. We quantified RBC concentration and reticulocyte count error structures and variances to perform statistical analyses. We performed two control experiments: one to control for blood sampling, another to control for housing conditions in order to prevent aggressive interactions. In the Results section we describe these experiments, the justification and formulation of the hypotheses we tested, and the statistical techniques we used to test them.

## Results

### Experimental data

#### Main experiment

Two of our mice (1 and 7) were observed in aggressive interactions after day 42. Both mice subsequently showed typical physiological signs of aggression (reduced RBC concentration and elevated reticulocyte proportion [26]). We therefore did not include the data for these two mice after day 42.

**Figure 1:**
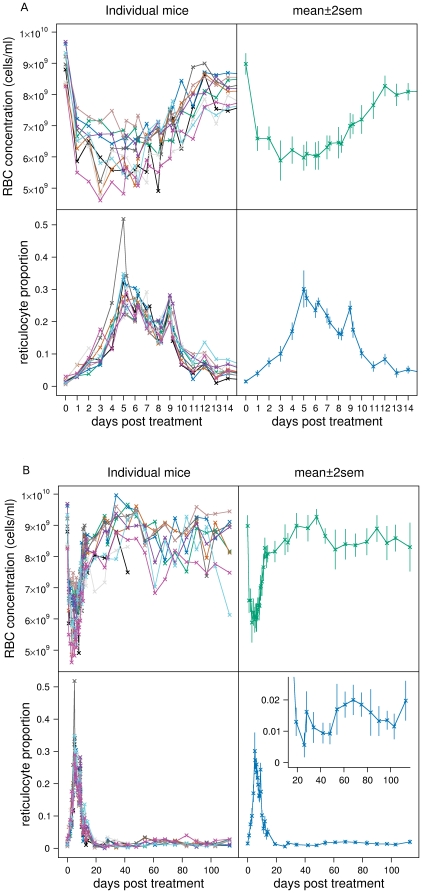
RBC and reticulocyte dynamics. Individual mouse data (left panels) and mean±2sem (right panels) of RBC concentration (top panels) and reticulocyte proportion (bottom panels) of the 10 mice in the main experiment for A: first 14 days, B: full 114 days. Inset expands reticulocyte proportion from days 15 to 114.

The top panels of Figure 1A show the RBC concentration dynamics over the first 14 days (left panel: individual mice, right panel: mean±2sem) The bottom panels of Figure 1A show the reticulocyte proportion dynamics over the first 14 days (left panel: individual mice, right panel: mean±2sem). Figure 1B shows the same over the full 114 days.

The RBC concentration dynamics show several interesting and unexpected features. There is an initial loss of about 30% of RBCs to about 6.5×10^9^ cells/ml in the first day. RBC concentration then drops at a slower rate for another 2 to 5 days depending on the mouse. There is a lot of variation in RBC dynamics across the mice which causes the mean dynamics to appear flatter than in the individual mice data. From day 6 to day 12 average RBC concentration increases by about 0.37×10^9^ cells/ml/day, but then abruptly slows by an order of magnitude to about 0.03×10^9^ cells/ml/day. What appears to be unusual about these dynamics is that this abrupt change occurs when RBC concentration is still below equilibrium (the change occurs at an average RBC concentration of about 8×10^9^ cells/ml whereas average equilibrium concentration is about 8.5×10^9^ cells/ml). Average RBC concentration then rises through its equilibrium value to become polycythemic reaching a maximum of about 9.3×10^9^ cells/ml on day 48. There is a period of cell loss between days 48 and 60 which is probably the clearance of the large cohort of RBCs induced by anaemia reaching their maximum lifespan. Thereafter RBC concentration appears to be in equilibrium.

The reticulocyte proportion dynamics are similarly intriguing. Over the first 3 days there is a gradual rise in average reticulocyte proportion from 1.5% to 10%. The rate of increase rises over the next 2 days culminating in an average peak proportion of 30% although there is a lot of variation between mice. Over the next 15 days reticulocyte proportion declines back to normal values but with a series of unusual aperiodic spikes on days 5, 6, 9, 12 and potentially day 14. Average reticulocyte proportion shows an unexpected drop below normal by day 26—unexpected because RBC concentration appears to reach equilibrium values at this time (see inset Figure 1B). This is followed by a rapid return to normal by day 28. It then drops below normal again until day 54 (approximately correlating with the slowly rising polycythemic RBC concentration). It stays higher than normal until about day 83 after which it returns to normal. On day 113 there is again a rise.

#### Epo concentration

**Figure 2:**
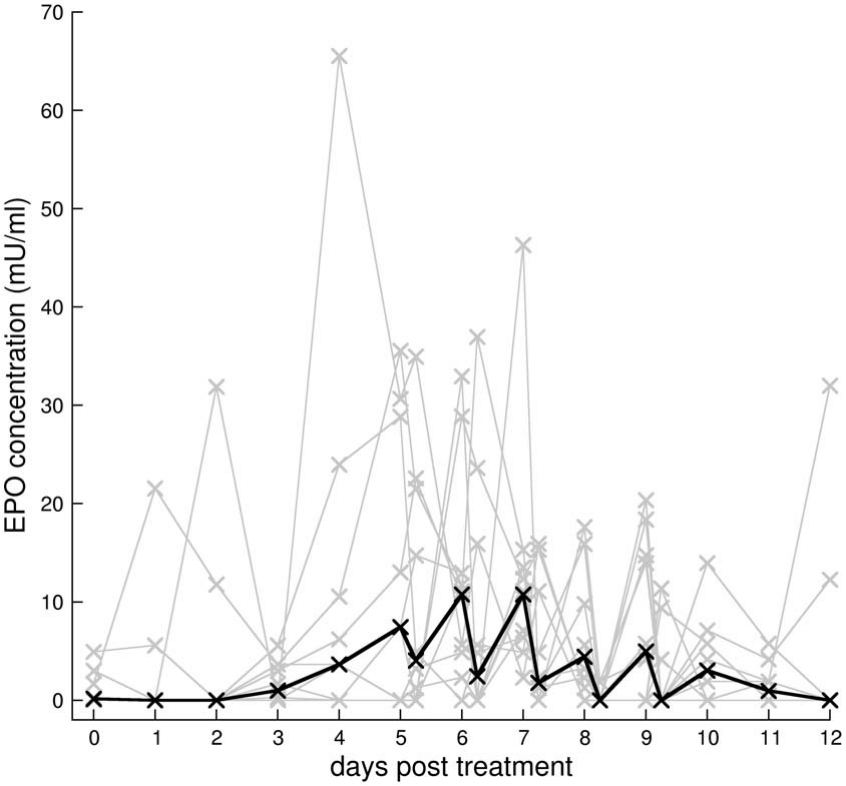
Serum Epo concentrations. Serum Epo concentrations for individual mice (grey lines) and median (black line) of main experiment. The two samples per day on days 5 to 9 suggest diurnal oscillations. Because we had to sample small volumes of blood (20 µl) our Elisa assay detection threshold is above normal Epo concentration, hence the many zeros in the data.

Figure 2 shows the individual mice Epo dynamics in grey and their median in black. Because we had to sample small volumes of blood (20 µl) our Elisa assay detection threshold is above normal Epo concentration, hence the many zeros in the data. The individual mice Epo data are difficult to interpret. However, several interesting patterns emerge from the aggregated data. Epo is generally undetectable until the third day, and then begins to rise over the next 3 days. From days 5 to 9, when we sampled twice per day, Epo concentration appears to exhibit diurnal oscillations with higher concentrations measured at 8am and lower at 3pm. Our data are consistent with mice being nocturnal, as humans have higher Epo concentrations in the afternoon compared to the morning [27]. After day 12, Epo concentration returns to undetectable levels. Oscillations may have occurred on days other than 5 to 9, but because we only sampled once per day diurnal oscillations would be undetectable.

To test if diurnal oscillations are indeed present, we performed a bootstrap analysis both within and across mice. For each mouse we counted the number of successive daily peaks and troughs in the Epo data (Table 1). We then bootstrapped without replacement each mouse's data 10^5^ times and counted the number of peaks and troughs in each simulated data set. This constructs a reference distribution of the number of peaks and troughs under the null hypothesis that Epo does not oscillate daily. Comparing the observed number of peaks and troughs to the reference distribution, we calculate a 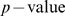 for the probability of observing at least as many peaks and troughs than was actually observed. These 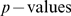 are given in Table 1 (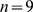). Except for mouse 5, the evidence for diurnal oscillations is weak. However, if we sum peaks and troughs across all mice there are 55 peaks and troughs in total. The 95% CI for the numbers of peaks and troughs across all mice under the null hypothesis is 24 to 45 (median 34), which gives a 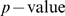 of 0.00051 (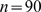), thus strongly suggesting that Epo does oscillate diurnally. This difference in 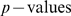 between tests on individual and aggregated data is due to the differences in statistical power of the tests which depend on sample size.

**Table 1:** Epo oscillations.

mouse	peaks+troughs	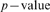
1	3	0.70
2	5	0.26
3	7	0.065
4	7	0.073
5	9	0.014
6	7	0.064
7	2	0.93
8	4	0.40
9	6	0.10
10	5	0.19
all	55	0.00051

Testing diurnal oscillations in Epo data.

#### Control experiments

One of the five mice in the group-housed, PHZ-treated control experiment, and one of the five mice in the group-housed, non-PHZ-treated control experiment had anomalous RBC and reticulocyte dynamics and were removed from the analysis.

**Figure 3:**
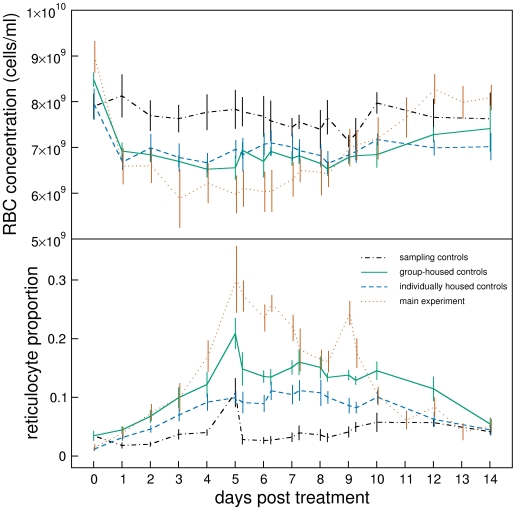
Control experiments. Mean±1sem of RBC concentration (top panel) and reticulocyte proportion (bottom panel) of main experiment (red dotted lines, same as in Figure 1) sampling controls (black dot-dashed lines), individually-housed, PHZ-treated controls (blue dashed lines) and group-housed, PHZ-treated controls (green solid lines). Lines are slightly offset from each other to reveal extent of error bars.

Group and individual housing had no significant effect on RBC concentration dynamics (compare green solid and blue dashed lines in Figure 3 top panel). However, individual housed mice had significantly lower reticulocyte proportions than group housed mice and did not exhibit reticulocyte spikes (bottom panel).

In the blood sampling control experiment mice were not given PHZ but were blood sampled as in the main experiment. Blood sampling had no effect on RBC concentration (black dot-dashed line in Figure 3, top panel). However, it produced a clear spike in reticulocyte proportion in three of the four mice on day 5, with potentially other spikes at other times in some mice (black dot-dashed line in Figure 3, bottom panel).

## Models

### Assessment of best fit models

**Figure 4:**
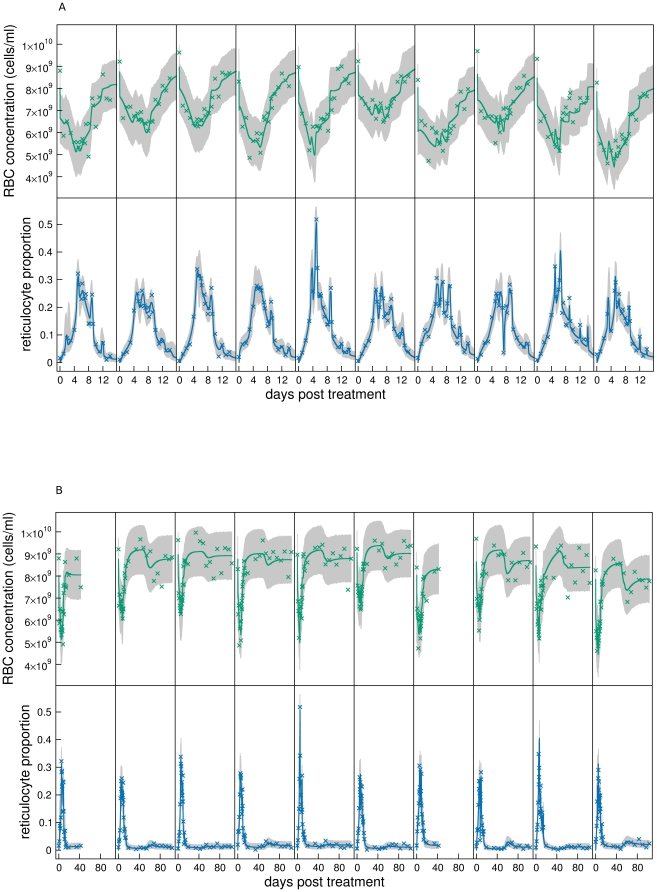
Model fits. Fits of best fitting model to RBC concentrations and reticulocyte proportions of individual mice of the main experiment. A: First 14 days, and B: all data. Grey regions are 95% posterior predictive intervals (95% of data should lie within this region), and solid lines are median solutions constructed from the 10^4^ samples of the posterior distribution.

Our best fitting model consisted of hypotheses A1, B1, C1, D1, and E1. The fits to all mice are shown in Figure 4 (A: first 14 days, B: all data, top panels: RBC concentration, bottom panels: reticulocyte proportion). The solid lines are the median fits, and the grey regions the 95% posterior predictive intervals.

**Figure 5:**
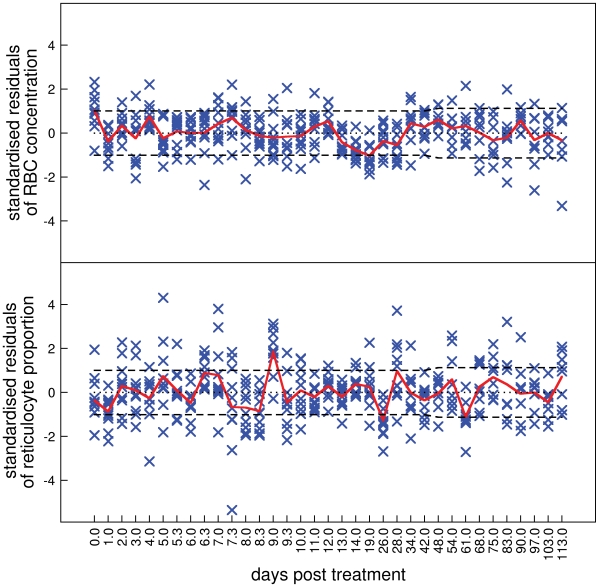
Standardised residuals. Assessment by standardised residuals of the model consisting of hypotheses A1, B1, C1, D1, E1 fitted to all data of the main experiment. Top panel: RBC concentration, bottom panel: reticulocyte proportion. Each cross represents the standardised residual of a time point for an individual mouse. These have an approximately normal distribution with mean 0 and variance 1. The solid line is the mean of the standardised residuals at each time point. The dashed lines define the approximate interval (Bonferroni corrected for multiple tests) within which we expect the mean to lie 95% of the time if the model were true. Note the nonlinear 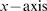.

More instructive plots for model assessment are the overlaid standardised residuals for RBC concentration (Figure 5, top panel, note nonlinear 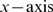) and reticulocyte proportion (Figure 5, bottom panel) of all mice in the main experiment. Inadequate model fits are suggested by extreme residuals and serial correlation between residuals.

The fits to RBC concentration are generally adequate but there are two causes for concern. The RBC concentration on day 0, i.e., just before PHZ treatment, is underestimated (Figure 5, top panel, 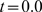). It is clear in Figure 1B that RBC concentration on day 0 is significantly higher (mean of 9×10^9^ cells/ml) than after about day 60 when presumably RBC concentration has returned to equilibrium (8.5×10^9^ cells/ml). Moreover, mean RBC concentration on day 0 in the control experiments (Figure 3) is lower than in the main experiment. These two pieces of evidence lead us to suggest that RBC concentrations on day 0 in the main experiment were higher than normal. This could be because of pre-experiment conditions or transportation, although we can only speculate on this. The second cause for concern is the serial correlation of residuals between days 13 and 61. This corresponds to the transition from anaemia to polycythemia and return to equilibrium.

The reticulocyte proportion data is not explained as well as for the RBC concentration data. The main causes for concern are an underestimation of the peak on day 9, and an overestimation on day 26. Between day 19 and 26 there is a significant, if albeit small, drop in reticulocyte proportion (Figure 1B, inset) that is not explained in our models by reduced production or release rate of reticulocytes caused by polycythemia. The model also overestimates reticulocyte proportion on day 61 when RBC concentration returns to equilibrium (Figure 1B). Aside from the possibility that sampling-influences on reticulocyte proportion exist and show temporal variation, we do not have any hypotheses to explain these discrepancies.

### Model comparison and analysis of hypotheses

In the following analysis we take as our baseline model the model consisting of hypotheses A1, B1, C1, D1 and E1 (one of the best fitting models). Then, taking each mechanism in turn, we replace its corresponding baseline hypothesis with another hypothesis of that mechanism. These models are fitted to the data and compared to the baseline model using Bayes factors (Table 2).

**Table 2:** Model comparison.

reticulocyte production rate		action of PHZ		reticulocyte release rate		RBC clearance		Reticulocyte spikes	
A2	12	B2	34	C2	1.1	D2	412	E2	40
A3	−1.8	B3	42	C3	22	D3	188	E3	186
A4	0.2	B4	40	C4	11				
A5	20								
A6	−1.8								

Comparison of each hypothesis against the best fitting model consisting of hypotheses A1, B1, C1, D1 and E1. Decibans >10 are strong evidence against a hypothesis (Equation 26).

#### Reticulocyte production rate

**Figure 6:**
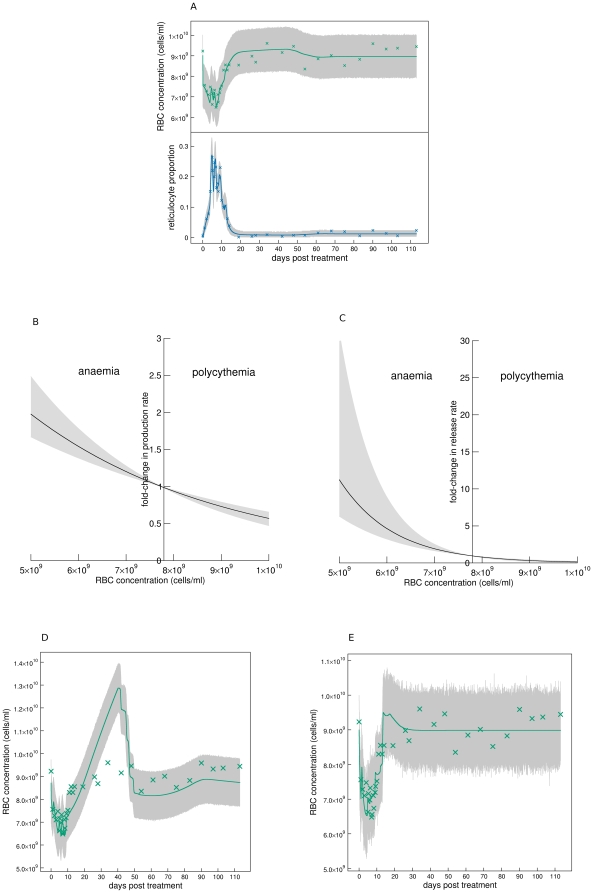
Hypothesis testing. A: Hypothesis A2: polycythemia not reducing production rate does not capture RBC and reticulocyte dynamics after day 60 (shown for mouse 6). B: Hypothesis A1: Production rate varies almost linearly with anaemia even allowing for an exponential relationship (shown for mouse 6). C: Hypothesis C1: reticulocyte release rate is exponentially related to anaemia (shown for mouse 6). D: Hypothesis D2: fixed RBC lifespan does not capture RBC dynamics. E: Hypothesis D3: unlimited RBC lifespan does not capture RBC dynamics.

There is strong evidence (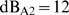) that polycythemia causes a reduction in reticulocyte production rate (hypothesis A1) rather than having no effect (hypothesis A2). This can be seen by comparing the dynamics of the two hypotheses. If polycythemia does not affect production rate, RBC concentration and reticulocyte proportion immediately return to equilibrium after the drop in RBC concentration after day 60 (see fit of mouse 6 in Figure 6A). However, if polycythemia does cause a reduction in production rate, the dynamics overshoot before returning to equilibrium (Figure 4B). The mean reticulocyte dynamics (Figure 1B) appear to support the latter rather then the former hypothesis, and this is consistent with experimental evidence [28],[29] as discussed in the section Production rate of bone marrow reticulocytes.

We cannot discriminate between a linear relationship between the difference in normal and anaemic RBC concentrations and reticulocyte production rate (A1), an exponential relationship (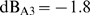) and a sigmoidal relationship (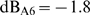). This is demonstrated in Figure 6B: over the range of RBC concentrations (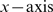) we observe for mouse 6, the fold-change in production rate (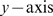) varies approximately linearly even for the model with an exponential relationship. Because we are still in the linear region of this response curve, the sigmoidal response does not improve the fit of the model.

We cannot discriminate between non-oscillatory (A1) and oscillatory production rate (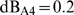). This does not mean that oscillatory production does not occur, just that any signal of oscillations is too weak to detect in our data.

There is no support for a time lag in the feedback between RBC concentration and reticulocyte production (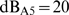).

#### Action of phenylhydrazine

**Figure 7:**
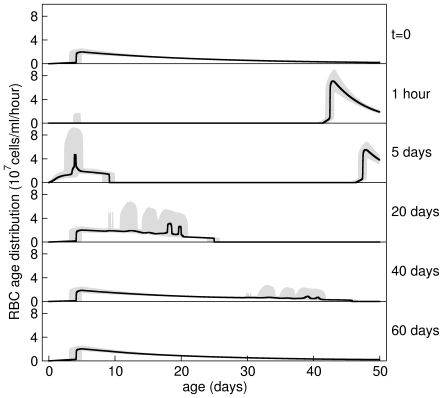
Predicted RBC age distributions. Prediction of circulating RBC age distribution just before, just after, and at various days after, PHZ treatment for mouse 6. The black line shows the estimated median age distribution and the grey region the 95% posterior predictive interval calculated from the samples of the posterior distribution of the model consisting of hypotheses A1, B1, C1, D1, E1. On day 0 the age distribution is in equilibrium made up of about 1% reticulocytes. All RBCs are aged with PHZ treatment causing an approximately 30% drop in RBC concentration, and a higher than normal clearance rate for about 7 days. Anaemia induces early release of reticulocytes from bone marrow (day 5) and increased reticulocyte production. Random clearance of RBCs prevents a large drop in RBC concentration about 50 days after PHZ treatment (day 40). The system returns to equilibrium after about day 60.

Hypothesis B1 states that RBCs present in the circulation at the moment of PHZ treatment are immediately aged. This is consistent with our data because it accounts for the initial loss of about 30% of RBCs within the first day due to ageing of older RBCs past their maximum lifespan 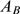 (see predicted dynamics of RBC age structure of mouse 6 in Figure 7). Hypothesis B1 also states that younger RBCs are aged more than older RBCs. This hypothesis is consistent with our data because it accounts for the reduction in RBC concentration from days 2 to 5 due to higher than normal clearance rate (see Figure 4A). (If no PHZ-induced lysis occurred during this time, RBC concentration would rise because production rate of reticulocytes would be greater than random clearance of RBCs.)

Hypothesis B2 states that RBCs present in the circulation at the moment of PHZ treatment lyse at a constant rate irrespective of their age. Thus this population decays exponentially and new RBCs entering the circulation are not aged by PHZ. This hypothesis is not supported (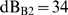).

Hypothesis B3 states that PHZ lyse all circulating RBCs at a rate proportional to its concentration and that concentration decays exponentially over time. This hypothesis is not supported (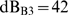).

A problem with hypotheses B2 and B3 is that they cannot account for the abrupt change in RBC loss between days 1 and 2. Hypothesis B4 overcomes this by adding an initial rapid loss to hypothesis B3. The addition of this mechanism, however, is also not supported as 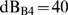).

#### Reticulocyte release from bone marrow

We cannot discriminate between polycythemia causing a reduction in reticulocyte release rate from bone marrow (C1) or having no effect on (C2) release rate (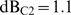).

An exponential (C1) rather than a linear (C3) relationship between the difference in normal and anaemic RBC concentrations and reticulocyte release rate is favoured (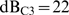). This is demonstrated in Figure 6C which shows the prediction of this relationship for mouse 6. However, for a linear relationship, when RBC concentration rises above the threshold 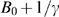 during polycythemia, release rate becomes 0 causing the reticulocyte proportion to be 0 until RBC concentration drops below this threshold; this is not observed in our data. Thus a linear relationship may be an adequate hypothesis, but its combination with polycythemia affecting release rate causes it not to be supported by the data. With no evidence for or against polycythemia causing a reduction in release rate, we must check the combined hypotheses of a linear relationship and polycythemia not affecting release rate. This hypothesis is not supported (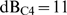), so we can conclude that an exponential relationship between the difference in normal and anaemic RBC concentrations and reticulocyte release rate is supported.

#### Clearance

**Figure 8:**
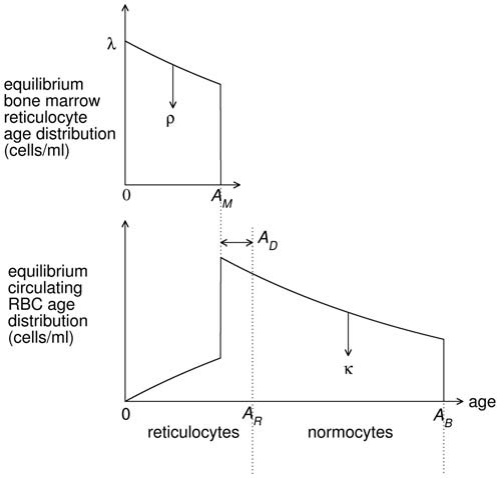
Schematic of RBC age distributions. Schematic of age distribution of reticulocytes in bone marrow and reticulocytes and normocytes in the circulation under normal equilibrium conditions. Reticulocytes are produced at a rate 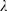 in bone marrow. These are released into the circulation at a rate 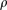. All reticulocytes are released by age 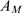, and have a maturation time of 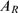 hours. RBCs are randomly cleared at a rate 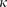 and have a maximum lifespan of 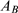 days. Not to scale.

Hypothesis D1 states that there is random clearance of RBCs until they reach their maximum lifespan (about 50 days) at which they are immediately cleared. This hypothesis gives age distributions as shown in Figures 7 and 8. This hypothesis is supported for two reasons. i) Under equilibrium conditions, input rate of RBCs from bone marrow is equally matched with clearance rate. The two mechanisms that clear RBCs are random clearance and reaching the maximum lifespan. Now, treatment with PHZ lyse all RBCs present at the time of administration within about a week (see Figure 7). The post-treatment cohort of RBCs only experience random clearance until they reach their maximum lifespan. Therefore, until this time, RBC input rate into the circulation is slightly higher than their clearance rate causing a slowly rising polycythemia which we observe in our data. If there were no random clearance of RBCs (hypothesis D2) their concentration would grow by about 0.5×10^9^ cells/ml each day (equal to 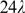) once RBC concentration had reached equilibrium at about day 12. We observe growth an order of magnitude lower than this at 0.03×10^9^ cells/ml/day. One might argue that the resulting polycythemia could cause a reduction in reticulocyte production rate to this level. But, to achieve a growth rate of 0.03×10^9^ cells/ml/day would require an order of magnitude reduction in reticulocyte production rate causing reticulocyte proportion to fall to about 0.15%. This though, is not consistent with our data which falls to just 1% during polycythemia. The fit of hypothesis D2 (shown in Figure 6D) confirms this, and so this hypothesis is not supported by the data (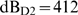). ii) If there is no immediate clearance of RBCs of around 50 days old (hypothesis D3) we do not observe a drop in RBC concentration at this time (Figure 6E). This hypothesis is not supported (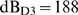).

#### Reticulocyte spikes

Hypothesis E3 tests if reticulocyte spikes can be produced intrinsically in our base model. This hypothesis is not supported (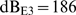). Sampling-induced reticulocytes must therefore be produced by some unknown mechanism not explicitly included in our model. We can test how these reticulocytes enter the erythropoietic system. Hypothesis E2 tests if spikes are produced by large transient increases in the production rate of reticulocytes in the bone marrow. This hypothesis is not supported (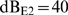) because the maturation age of reticulocytes is about 2–4 days, so spikes would be observed for that length of time, instead we observe spikes lasting for less than a day. This observation led us to suggest hypothesis E1 in which sampling-induced reticulocytes enter directly into the circulation.

### Parameter estimates

#### Main experiment

The marginal distribution of the standard deviation of RBC measurement error 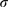, is 0.53×10^9^ cells/ml with a 95% CI of 0.49 to 0.58×10^9^ cells/ml.

**Figure 9:**
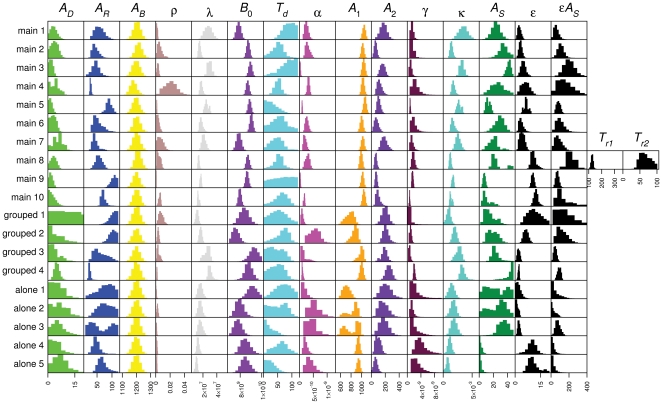
Parameter estimates. Marginal distributions of parameters of model consisting of hypotheses A1, B1, C1, D1, and E1 for main, and housing control experiments.

Figure 9 shows the marginal distributions of the parameters of the model consisting of hypotheses A1, B1, C1, D1 and E1. The base model (Equations 1–6) has six parameters, the reticulocyte maturation time 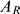, the maximum reticulocyte residence time in bone marrow 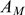 (equal to 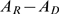), the normal reticulocyte production rate 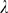, the normal reticulocyte release rate 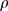, the circulating RBC clearance rate 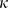, and their maximum lifespan 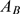.

The 95% CIs of the marginal distributions of 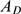 contain 0 and do not go above about 10 hours (for the main experiment mice at least, which have more data and therefore give more accurate parameter estimates). This suggests i) that normocytes do not remain in bone marrow (which would occur if 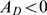 which we allowed it to do), and ii) that, in normal conditions, most reticulocytes are released as, or at most about half a day before, they mature. This is similar to sheep [30], but not humans in which reticulocytes appear to be released about a day before they mature [31].

For most mice the distribution of 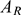 is not well defined, taking values between 2 and 5 days. It also exhibits strong negative correlation with 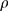 (not shown) because both parameters determine the concentration of circulating reticulocytes under normal conditions (Equation 23). So a change in 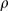 can be compensated for with a change in 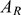.

The marginal distributions of 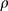 exhibit a large variation across mice from 7.9×10^−4^ (95% CI: 7.1×10^−4^, 8.5×10^−4^) hr^−1^ for mouse 9 to 0.021 (0.015, 0.026) hr^−1^ for mouse 4. Taken together, the broad estimates in 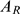 and 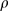 suggest that our data do not allow us to precisely determine the age distribution of reticulocytes in bone marrow and the circulation. More precise priors on one of these parameters would improve the precision of the other. We have not been able to find estimates for either 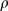 or 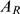 in mice in the literature. In humans 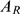 is thought to be about 4 days [32], and in rats about 3 days [18].

We took a narrow prior for maximum RBC lifespan (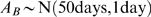) to improve identifiability of 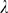 and 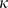. The mean of 50 days corresponds roughly to the drop in RBC concentration and previous estimates [33]. There is very little difference between the marginal distributions of 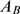 and their prior. Therefore our data poorly identify 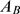.

The marginal distributions for normal RBC concentration 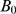, although variable between mice, are precise. It is interesting that the mice observed in aggressive interactions (1 and 7) have low estimated 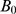.

The marginal distributions of reticulocyte production rate 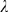 and RBC clearance rate 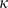 are correlated because reticulocyte production rate must match RBC clearance rate in equilibrium. The marginal distributions of 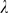 vary between 1.31×10^7^ (1.25×10^7^, 1.44×10^7^) cells/ml/hr for mouse 10 and 2.94×10^7^ (2.77×10^7^, 3.13×10^7^) cells/ml/hr for mouse 3. The marginal distributions of 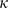 are, in general, quite broad, varying between 0.00122 (0.00112, 0.00132) hr^−1^ for mouse 8 and 0.00347 (0.00295, 0.00395) hr^−1^ for mouse 1. Mouse 1 has a high modal clearance rate, and this is reflected in its flat RBC concentration after day 12 (Figure 4B) rather than the increasing concentration seen in the other mice. This is probably partly an artifact of the shorter time series for this mouse. Mouse 3 has a similarly high clearance rate. Again this is reflected in its relatively flat RBC concentration after day 12. In other modelling studies RBC clearance rate is assumed to be around 0.02 to 0.025 day^−1^ (0.001 hr^−1^) because RBC lifespan in mice is about 40 to 50 days.

The modal value of the time to delay before increased production rate 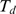, is about 2.5 days. This corresponds to just before the rapid increase in reticulocyte proportions are observed (Figure 1) and when Epo concentrations rise above detection (Figure 2). However, it appears that a delay is only required to adequately explain the data of some of the mice; the 95% CI of 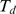 in the majority of mice contains 0. This is probably because only 2 or 3 data points provide estimates for 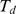.

The marginal distributions for the scaling factor between anaemia and fold-increase in RBC production rate 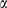 vary between 0.28×10^−10^ (0.21×10^−10^, 0.37×10^−10^) (cells/ml)^−1^ for mouse 3, and 3.3×10^−10^ (3.0×10^−10^, 3.5×10^−10^) (cells/ml)^−1^ for mouse 4.

The PHZ-induced change in age of RBCs of age 0, 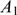, are well defined within mice and vary between 41 and 45 days across mice. The PHZ-induced change in age of RBCs of age 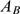, 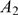, is less well defined; for most mice it is between 2 and 9 days with a mean of roughly 3 days.

The marginal distributions for the scaling factor between anaemia and fold-increase in reticulocyte release rate 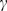 vary between 0.70×10^−9^ (0.65×10^−9^, 0.74×10^−9^) (cells/ml)^−1^ for mouse 10 and 2.24×10^−9^ (1.74×10^−9^, 2.93×10^−9^) for mouse 4.

The maturation time of sampling-induced reticulocytes in the circulation 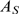 is quite variable, from as little as 4 hours in mice 9 and 10, up to about 2 days in other mice. The marginal distribution for mouse 4 is quite broad, most likely reflecting the lack of reticulocyte spikes for this mouse (Figure 4B). The fold-increase in sampling-induced reticulocyte production rate 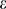 is quite broad varying between 2.5 (1.9, 3.2) for mouse 1 and 12 (11, 13) for mouse 10.

#### Housing control experiments

The marginal distributions of the PHZ-treated control mice are shown in Figure 9. Most of the parameters have similar distributions as the main experiments, although some are slightly wider due to fewer data for these mice. The most striking difference in parameter estimates is the PHZ-induced ageing parameter 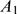.

Initially we used the same priors for the control experiments as we did for the main experiment. We found that RBC clearance rate 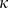, was not well defined in control mice (data not shown) because the RBC dynamics had not returned to equilibrium by the end of these experiments. This affected the estimation of the PHZ-induced ageing parameters 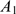 and 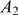 because these depend on the equilibrium RBC age distribution which depends on 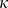. A potential difference between the main and the control experiments could have been the efficacy of the PHZ administered because these experiments were done with different batches of PHZ. Such a difference could affect the PHZ-induced ageing of RBCs. Therefore, to be able to compare PHZ-induced ageing between the main and control experiments we based the prior of 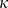 for the control experiments on its estimated marginal distributions from the main experiment. The prior we chose was N(0.0015, 0.0005). The posterior estimates shown in Figure 9 clearly demonstrate that 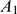 is smaller for most mice in the control experiments. We therefore suggest that the efficacy of the PHZ administered in these experiments was weaker than for the main experiment. This could explain why mice in the main experiment were more anaemic and had higher reticulocyte proportions than control mice (Figure 3).

Parameters 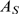 and 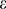 determine the duration and magnitude of the spikes in reticulocyte proportion. For mouse 4 in the main experiment and all the individually housed mice, the 95% CIs of the product of these two parameters contains 0. This correlates with the observation that these mice showed weak or no spikes.

### An outlier in mouse 8?

During day 7, the reticulocyte proportion in mouse 8 dropped dramatically to almost normal levels (0.036%, Figure 4A), before rapidly rising again. This could be because the blood smear was mixed up with another, although we are certain this did not occur. It could be an anomalous count, although the probability of counting just 18 reticulocytes in 500 RBCs instead of around 117 reticulocytes (which is the average of the other 9 mice at this time) is unlikely (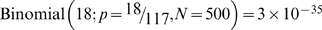). Or it could be a real phenomenon. None of our models capture this behaviour. Instead we have assumed in the above analysis, and for this mouse only, that the release rate of reticulocytes from time 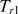 to 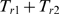 returns to baseline 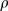. The marginal distributions for 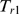 is 126 (121, 131) hrs and for 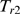 64 (54, 78) hrs (Figure 9). We have tried allowing a drop in production rate but with no success. We have no explanation for why a temporary return to baseline release rate may have occurred only in this mouse.

## Discussion

The aim of this paper was to synthesise ideas from previous experimental data and the mathematical modelling literature with new data in order to test old and new hypotheses about mechanisms that govern reticulocyte and RBC age structure and population dynamics in bone marrow and the circulation of mice. In contrast to previous model fitting studies we quantified measurement error. This allowed us to perform model fitting, model testing, model comparison and parameter estimation under a Bayesian framework. We tested multiple hypotheses that reflect uncertainty in erythropoietic mechanisms. Some of the hypotheses do not adequately explain the data and others do. So although we can rule out certain hypotheses, we are left with some that our data cannot discriminate between. This therefore suggests which mechanisms should be explored with further experiments and modelling.

The picture of the erythropoietic system emerging from this synthesis of data and modelling is as follows. Under normal conditions (see Figure 8) reticulocytes are produced at about 10^7^ cells/ml serum/hour in bone marrow. Reticulocytes mature for between 2–4 days with limited release (with rate constant of about 10^−3^ hr^−1^) into the circulation (we estimate about 2–5% of reticulocytes are in the circulation with the rest in bone marrow). Once matured into normocytes, any cells remaining in the bone marrow are immediately released into the circulation. RBCs undergo random clearance with a rate constant of about 0.002 hr^−1^ independent of age, which causes a negative exponential age distribution. On reaching about 50 days old there is rapid clearance of any remaining RBCs (we cannot estimate this rate with our data).

We postulate that treatment with a single dose of PHZ may age rather than kill RBCs. Older RBCs are aged past their maximum lifespan and are immediately cleared causing a rapid loss of RBCs within 24 hrs. Younger RBCs, which are affected more than older RBCs by PHZ, are not immediately cleared, but cause a higher than normal clearance rate for several days until all are lost (see upper three panels in Figure 7). Serum Epo response is delayed for about 3 days, and may correlate with a delay in increased production of reticulocytes in some mice. We have also discovered that Epo appears to exhibit pronounced diurnal oscillations at least during anaemia, but we cannot tell if this causes oscillations in reticulocyte production rate. Production rate of reticulocytes is linearly and negatively correlated with RBC concentration—at least across the range of concentrations we observed. On treatment with PHZ, release rate of reticulocytes from bone marrow increases immediately and dramatically. We estimate that at maximum anaemia, between 35 to 95% of reticulocytes were in the circulation. After about 12 days of increased production, RBC concentration overshoots normal levels causing polycythemia. This results in a lower than normal reticulocyte production rate causing a lower than normal reticulocyte proportion. Normal equilibrium conditions are restored after the PHZ-induced cohort of RBCs reach the end of their lifespan. Blood sampling caused aperiodic and pulsed release of reticulocytes into the circulation in mice housed in groups but did not in mice housed individually. Reticulocytes comprising spikes appear to have a different developmental pathway than normal reticulocytes because their mechanism of release into the circulation is different.

Erythropoietin mediates the negative feedback between haemoglobin concentration in the circulation and apoptosis of erythroid progenitor cells that develop into cell-cycle arrested reticulocytes [34]. We initially included erythroid progenitors in our model and assumed that they were irreversible committed to differentiate into reticulocytes. Under this assumption, we found that all the parameters that governed their age distribution were unidentifiable given the data we had. All we were able to quantify was the rate at which erythroid progenitors differentiate into reticulocytes in bone marrow (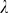) and how this rate is modified by RBC concentration (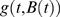). Including erythroid progenitors in the model did not improve its fit. In contrast, [9] assume that erythroid progenitors take 4 days to mature but do not estimate its value from model fitting, and [15] estimate their maturation time in rats to be 43 hrs with a coefficient of variation of 7.5% by fitting to data.

Although our model can adequately explain most of the data, where it does not, raises some interesting questions. For example, by what mechanism does blood sampling induce spikes in reticulocyte counts? What causes the temporal pattern of spikes and why were they synchronous in the ten main experimental mice but not in the control mice? Why were spikes less apparent and reticulocyte proportion lower in individually housed mice compared to grouped mice? What caused the return to baseline release rate in mouse 8? Why is there a 3 day delay in increased serum Epo concentration? Also our model does not appear to adequately explain RBC and reticulocyte dynamics between days 13 to 61 when RBC concentration overshoots its normal levels and becomes polycythemic. We hope to address theses questions with further experimental and modelling work.

## Methods

### Ethics statement

All experimental procedures were regulated and carried out under the U.K. Home Office Animals (Scientific Procedures) Act, 1986.

### Experiments and data collection

Adult male MF1 mice were used for all experiments for two reasons: their large size reduces the effect of blood sampling their erythropoietic state, and their docile and non-aggressive nature reduces the chance that stressful interactions when housed in groups affects RBC and reticulocyte dynamics [35],[36].

At each sampling time point we took 2 µl blood to measure RBC concentration, we took a blood smear with approximately 1 µl blood to measure reticulocyte proportion and we took 20 µl blood to measure serum Epo concentration. We diluted 1∶40,000 the 2 µl blood sample and measured RBC concentration by coulter counter. We stained blood smears with 10% Giemsa in pH 7.2 buffer and estimated reticulocyte proportion by counting the number of reticulocytes observed in at least 500 RBCs by light microscopy.

We measured Epo concentration using an Elisa adapted from [37] to measure Epo concentration from small blood samples and thus permit repeat sampling of each mouse. We collected 20 µl of blood in 5 µl of heparin for each sample point. We centrifuged this at 13,000 rpm for 3 minutes, and collected and stored the serum at −80°C. We prepared the Elisa plates by adding 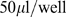 of 4 µg/ml rat anti-mouse Epo IgG1 antibody (BD, 554651) before incubation at 4°C overnight. We washed the plates with 0.1% Tween 20 in PBS and blocked them with 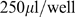 of 1% Bovine Serum Albumin (BSA) for 1 hr at room temperature. We loaded the plates with rmEpo (Roche, 112769640010) standards in a 2× dilution series (500-0.98 mU/ml). We defrosted the serum samples and diluted 20 µl of each in 80 µl of blocking buffer which we then used to make a 2× dilution series for each sample. We left the plates to incubate overnight at 4°C. After washing 3 times, we added, at 10 µg/ml, 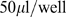 of polyclonal rabbit anti-hEpo antibody (R&D, AB286NA) and incubated them at room temperature for 1 hr before washing a further 3 times. We incubated the plates with 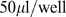 of HRP-conjugated goat anti-rabbit IgG (Bio-Rad, 1708241) at a 1∶3000 dilution in blocking buffer at room temperature for 1 hr. We detected the bound conjugate by adding 0.1ml/well of ABTS substrate with 0.036% hydrogen peroxide and developed for 20 minutes before reading at wavelength 405 nm with 595 nm as reference. We obtained the concentrations of Epo in the serum samples by comparison with values of the rmEpo standard curve.

The main experiment consisted of 10 mice treated with 40 mg/kg PHZ intraperitoneally and housed together. Their blood was sampled 35 times: from just prior to PHZ administration to 113 days post treatment. We measured RBC concentration and reticulocyte proportion for each sampling point and measured Epo concentration up to day 42. As a temporal control for blood sampling, we took the 20 µl of blood required for Epo measurement at each sample point for all mice in all experiments whether or not it was used for Epo measurement. As our trial experiment on PHZ-treated MF1 mice demonstrated fast reticulocyte dynamics from days 5 to 9 post PHZ treatment we sampled twice per day during this period to ensure improved model discrimination. We took samples daily for days 0 to 4 inclusive and days 10 to 14 inclusive and weekly for another 13 weeks. We took samples at 9am, and, for twice daily sampling, at 3pm. We carried out additional experiments to investigate any effects of blood sampling and group housing on erythropoietic dynamics. This involved monitoring reticulocyte proportion and RBC concentration in three groups of five mice: group-housed, non-PHZ-treated mice, group-housed, PHZ-treated mice, and individually-housed, PHZ-treated mice. Sampling frequency was the same as for the main experiment except that samples were only collected up to day 14.

### Models and hypotheses

We use a two-compartment, continuous-time, age-structured formalism for our models. The two compartments are bone marrow and the circulation. We model reticulocyte concentration in bone marrow and the circulation (with conversion to a proportion of total RBC concentration for model fitting) and RBC concentration in the circulation. In this section, we describe the mathematical formulation of the base model. We then consider a range of competing hypotheses that reflect uncertainty in the mechanisms of the erythropoietic system.

The youngest bone marrow cells we consider are erythroid progenitor cells that have just differentiated into cell-cycle arrested reticulocytes (Figure 8). We define their age to be 0. Under normal equilibrium conditions we assume that their production rate is 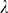 with units of cells/ml blood serum/hr. We assume that reticulocyte maturation time is 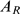 hours.

The production rate of reticulocytes is mediated by erythropoietin (Epo), a glycoprotein hormone produced in the kidney and other organs [38],[39]. Epo controls erythroid progenitor growth by retarding their DNA breakdown and preventing apoptosis [34]. It was our intention to construct models that related reticulocyte production (from erythroid progenitors) to serum Epo concentration which in turn was related to the difference between normal and anaemic circulating RBC concentrations. However, on analysis of our Epo data, we concluded that we could not have absolute confidence in the precision of the Elisa data, possibly due to the small volumes of blood analysed and the accuracy of interpolation from our standard curve. Moreover, we had no quantitative estimate of the experimental error in Epo concentration so we could not assess the adequacy of a model fit to it. Instead we directly relate reticulocyte production rate to the difference between normal and anaemic circulating RBC concentrations and neglect serum Epo concentration as an intermediate regulator.

We therefore assume that under anaemic conditions reticulocyte production rate 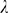, is multiplied by a function 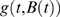 which is the fold-change in reticulocyte production rate compared to normal. Time since PHZ administration is 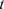 with units of hours and 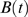 is the circulating RBC concentration at time 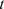 with units of cells/ml.

In normal conditions in humans, reticulocytes mature for about 3 days in bone marrow, are released into the circulation, and complete maturation within a day [32]. As RBC concentration falls, maturation time remains constant at about 4 days but residence time in bone marrow decreases linearly with RBC concentration [32]. Other studies have demonstrated that reticulocytes in humans, sheep and mice are released early from bone marrow in phlebotomy-induced anaemia [13],[16],[32] and intravenous dosing with recombinant human Epo [31].

Immature and mature reticulocytes can be differentiated by their RNA content because they lose RNA as they mature. Data from humans [31] show that, in normal conditions, about 5% of reticulocytes in the circulation are immature (using the definition of maturity in [31]) and 95% are mature. Stimulus of erythropoiesis, in this case by an injection of Epo, caused an immediate release of immature reticulocytes from bone marrow into the circulation. There was no increase of mature reticulocytes for another 3 days. This suggests the inclusion of the following two processes in our model. i) In normal conditions, most immature reticulocytes remain in bone marrow with some small amount of migration into the circulation. ii) Mature reticulocytes are released into the circulation before maturation into normocytes. We formulate these processes as follows. In normal conditions we assume that bone marrow reticulocytes are released into the circulation at a rate 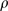. As for reticulocyte production rate, we model the direct relationship between the difference in normal and anaemic RBC concentrations and release rate, ignoring the intermediate regulation by Epo. Thus we assume that 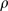 is modified under anaemic conditions by the function 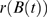 which is the fold-change in reticulocyte release rate compared to normal. We assume that there is a maximum residence time 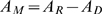, of cells in the bone marrow. If 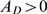 all reticulocytes exit the bone marrow before maturation, and if 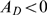 reticulocytes mature into normocytes before entering the circulation Figure 8.

Let 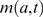 be the age distribution of bone marrow reticulocyte (plus normocyte if 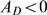) concentration with units of cells/ml/hour. The partial differential equation describing its rate of change is given by
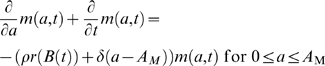
(1)where 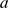 is cell age. The left-hand-side (LHS) of Equation 1 describes cell ageing, the first term on the right-hand-side (RHS) describes release of cells between ages 0 and 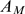 from bone marrow into the circulation and the second term (a Dirac-delta function) describes release of any remaining cells at age 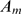.

Let 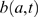 be the age distribution of circulating RBC concentration (reticulocytes plus normocytes) with units of cells/ml/hour. RBCs are cleared by phagocytosis in the spleen, liver and bone marrow. Cohort labelling with radioiron in mice suggests that RBCs are randomly cleared at an age-independent rate, with negligible RBCs surviving more than about 50 days [33]. In humans, however, there appears to be little clearance before about 120 days with rapid clearance thereafter [40]. We combine these two pieces of evidence and assume that, up to 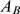 days old (about 50 days), RBCs are randomly cleared at a relatively slow rate 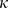. After that, clearance rate accelerates. With our data we can estimate 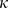, but we cannot quantify the acceleration in clearance rate. We therefore assume that RBCs have a maximum lifespan of 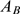 hours; in other words, any RBCs that reach the age of 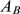 are immediately cleared.

The partial differential equation describing the rate of change of circulating RBC age distribution is given by
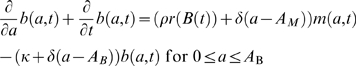
(2)The LHS of Equation 2 describes RBC ageing, the first two terms on the RHS describe release of bone marrow cells into the circulation, and the last two terms describe RBC random clearance and immediate clearance at their maximum lifespan respectively.

The boundary conditions for Equations 1 and 2 at 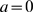 are the production rate of bone marrow reticulocytes and 0 (because RBCs enter via the bone marrow) respectively, i.e. (see Figure 8),
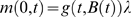
(3)

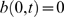
(4)Note that, in normal conditions 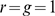.

The concentrations of circulating reticulocytes 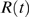, and circulating RBCs 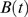 (reticulocytes plus normocytes), are
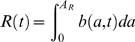
(5)

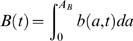
(6)All variables, functions and parameters are listed in Table 3. In the following sections we formulate the hypotheses we will test.

**Table 3:** Variables and parameters used in the mathematical models.

**Independent variables**		
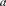	age	Hr
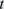	time	Hr
**Dependent variables**		
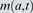	bone marrow cell age distribution	cells/ml/hr
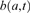	circulating RBC age distribution	cells/ml/hr
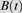	circulating RBC concentration	cells/ml
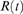	circulating reticulocyte concentration	cells/ml
**Functions**		
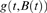	anaemia-induced fold-change in reticulocyte production rate over normal	
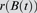	anaemia-induced fold-change in reticulocyte release rate over normal	
**Parameters**		
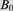	normal circulating RBC concentration, equal to 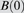	cells/ml
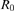	normal circulating reticulocyte concentration, equal to 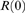	cells/ml
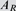	reticulocyte maturation time	hr
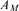	maximum residence time in bone marrow	hr
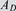	equal to 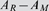	hr
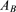	maximum RBC lifespan	hr
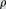	normal bone marrow reticulocyte release rate into circulation	hr^−1^
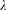	normal bone marrow reticulocyte production rate	cell/ml/hr
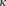	clearance rate of circulating RBCs	hr^−1^
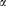	scaling factor between difference in normal and anaemic RBC concentrations and reticulocyte production rate	(cells/ml)^−1^
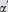	maximum fold-change in reticulocyte production rate over normal	
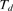	delay from PHZ treatment to increased reticulocyte production rate	hr
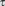	time lag in reticulocyte production rate	hr
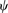	amplitude of oscillations of reticulocyte production rate	
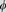	initial phase of oscillations of reticulocyte production rate	radians
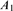	PHZ-induced change in age of RBCs of age 0	hr
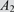	PHZ-induced change in age of RBCs of age 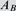	hr
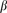 , 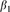	PHZ-induced RBC lysis rate	hr^−1^
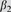	decay rate of PHZ	hr^−1^
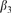	proportion of RBCs lysed just after PHZ treatment	
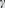	scaling factor between difference in normal and anaemic RBC concentrations and reticulocyte release rate	(cells/ml)^−1^
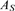	sampling-induced reticulocytes' maturation time	hr
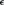	fold-increase in sampling-induced reticulocytes' production over normal	
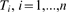	time when reticulocyte spike 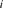 starts	hr
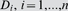	duration of reticulocyte spike 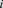	hr
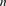	number of reticulocyte spikes	

#### Production rate of bone marrow reticulocytes

Epo production is known to increase exponentially with falling RBC concentration [41],[42], but the functional relationship between serum Epo concentration and production rate is not known. Sigmoidal [1],[9] and linear [11] responses have been used in previous models. We considered three hypotheses which relate RBC concentration to reticulocyte production rate: exponential (hypothesis A1), linear (hypothesis A3) and sigmoidal (hypothesis A6). See below for their formulation.

When oxygen supply exceeds demand, as for instance during repeated transfusions of RBCs causing polycythemia (higher than normal RBC concentration), circulating reticulocyte concentration decreases due to production of fewer reticulocytes in bone marrow [28],[29]. We consider two hypotheses: polycythemia reduces (hypothesis A1), or has no effect on (hypothesis A2), production rate.

There is evidence of diurnal oscillations in circulating reticulocyte concentration and mitotic activity in bone marrow in rats and mice [43]. Our data and other work [44] suggest diurnal oscillations in Epo concentration in mice. It is therefore suggestive that diurnal oscillations in Epo could cause oscillations in production rate. We therefore tested two hypotheses: with (hypothesis A4), and without (hypothesis A1), diurnal oscillations in production rate.

In our data, two pieces of evidence suggest there may be a delay of several days from administration of PHZ to increased production rate: the rapid rise in reticulocyte proportion around day 3 (Figure 1A and 1B), and Epo concentration generally below detectability until about day 3 then rapidly rising (Figure 2). Hypothesis A1 tests this idea by only allowing increased production after some time 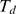. Hypothesis A5, however, tests another idea that may explain the data: production rate at time 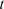 depends on RBC concentration at some earlier time 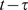. This idea has been used in other modelling studies (e.g., [20]). The mathematical formulations of all these hypotheses are detailed below.

**Hypothesis A1** Increased reticulocyte production is delayed after PHZ treatment. Production rate depends instantaneously and exponentially on the difference between normal and anaemic RBC concentrations.

This means 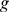 (the anaemia-induced fold-change in reticulocyte production rate over normal) in Equation 1 has the form
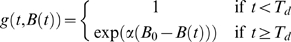
(7)where 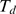 is the delay between PHZ administration and increase in production rate, 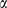 is a scaling factor and 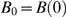, the normal RBC concentration.

**Hypothesis A2** As hypothesis A1, but production rate remains normal during polycythemia (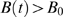).

This means 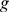 has the form
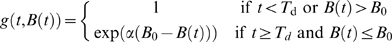
(8)


**Hypothesis A3** As hypothesis A1, but production rate depends linearly on the difference between normal and anaemic RBC concentrations.

This means 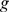 has the form
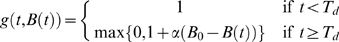
(9)The maximum value is taken to prevent production rate becoming negative during polycythemia.

**Hypothesis A4** As hypothesis A1, but reticulocyte production rate exhibits diurnal oscillations

This means 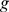 has the form
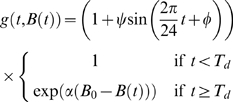
(10)where 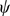 is the amplitude and 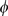 the initial phase of the oscillations.

**Hypothesis A5** As hypothesis A1, but with a lag in the feedback between reticulocyte production rate and RBC concentration instead of a delay from PHZ administration to increased production rate.

This means 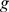 has the form
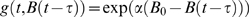
(11)where 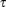 is lag time.

**Hypothesis A6** As hypothesis A1, but production rate depends sigmoidally on the difference between normal and anaemic RBC concentrations.

This means 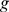 has the form
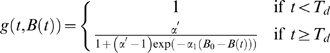
(12)where 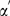 is the maximum fold-change in reticulocyte production rate over normal.

#### Effect of phenylhydrazine

Phenylhydrazine (PHZ) has been used for over 100 years to induce haemolytic anaemias in experimental animals [25] through RBC lysis. Remarkably, studies on whether PHZ alters the age distribution of RBCs have not been previously undertaken. We test four hypotheses for the action of PHZ on circulating RBCs; we assume it has no effect on bone marrow reticulocytes.

It has been shown in rabbits that PHZ is eliminated slowly from the body; 60% of a 50 mg/kg dose is removed in 10 days [45]. However, it is unknown if PHZ lyse only those RBCs present in the circulation when it is administered (hypothesis B2) or if in addition it lyses RBCs that enter the circulation after it is administered (hypothesis B3). For hypothesis B2 we assume that RBCs are lysed at a constant rate, and for hypothesis B3 we assume that lysis rate is proportional to PHZ concentration which decays exponentially over time.

These two hypotheses cause a smooth, exponential decay in RBC concentration. Our data suggest, however, an abrupt change from a fast to a slow lysis rate sometime before 24 hrs post PHZ administration (Figure 1A). We test this hypothesis (hypothesis B4) by removing a proportion of RBCs from the circulation just after PHZ administration.

PHZ is known to react with haemoglobin to produce free radicals that cause peroxidation of RBC lipid membranes leading to lysis [46]. This led us to consider the hypothesis that PHZ does not kill RBCs outright, but prematurely ages them (hypothesis B1). In addition, we consider the hypothesis that PHZ might age younger RBCs more than older RBCs, perhaps because younger RBCs are more metabolically active and thus more vulnerable to oxidative damage. The mathematical formulations of these hypotheses are detailed below.

**Hypothesis B1** RBCs present in the circulation when PHZ is administered are prematurely aged. RBCs entering the circulation after PHZ treatment are not aged by PHZ. The change in a RBC's age is linearly correlated with its age at time of treatment.

Just after 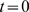, RBC ages are transformed to
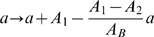
(13)where 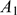 is the change in age of RBCs of age 0, 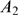 is the change in age of RBCs of age 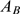, and 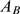 is the maximum RBC lifespan. Any RBCs aged past the maximum lifespan are immediately cleared.

**Hypothesis B2** RBCs present in the circulation when PHZ is administered lyse at a constant rate. RBCs entering the circulation after PHZ treatment are not aged.

Equation 2 is modified by addition of the term
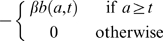
(14)where 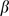 is the PHZ-induced lysis rate.

**Hypothesis B3** All RBCs in the circulation (pre and post PHZ treatment) lyse at a rate proportional to PHZ concentration which itself decays exponentially.

Equation 2 is modified by addition of the term
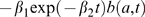
(15)where 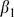 is the initial PHZ-induced lysis rate, and 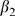 the decay rate of PHZ.

**Hypothesis B4** As hypothesis B3, but with immediate lysis of a proportion of RBCs present when PHZ is administered.

Equation 2 is modified as for hypothesis B3. In addition, just after 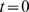, RBC age distribution is transformed to
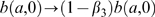
(16)where 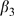 is the proportion of RBCs lysed just after treatment.

#### Reticulocyte release from bone marrow

We test if release rate is linearly (hypothesis C3) or exponentially (hypothesis C1) related to the difference in normal and anaemic RBC concentrations. We test if polycythemia reduces (hypothesis C1) or has no effect on (hypothesis C2) release rate. Finally we test the combined hypotheses of a linear relationship and polycythemia having no effect on release rate (hypothesis C4).

**Hypothesis C1** Reticulocyte release rate from bone marrow depends instantaneously and exponentially on the difference between normal and anaemic RBC concentrations.

This means 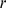 (the anaemia-induced fold-change in reticulocyte release rate over normal) in Equations 1 and 2 has the form
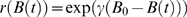
(17)where 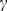 is a scaling factor.

**Hypothesis C2** As hypothesis C1, but polycythemia does not reduce release rate.

This means 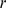 has the form
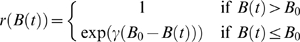
(18)


**Hypothesis C3** As hypothesis C1, but release rate depends linearly on the difference between normal and anaemic RBC concentrations.

This means 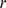 has the form
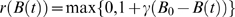
(19)The maximum is taken to prevent the release rate becoming negative during polycythemia.

**Hypothesis C4** As hypothesis C3, but polycythemia does not reduce release rate.

This means 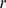 has the form
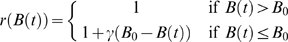
(20)


#### Clearance of circulating RBCs

We wish to test if RBCs are cleared randomly and independently of age (hypothesis D3), or if they are all cleared at a fixed age (hypothesis D2), or if they are cleared randomly up to a fixed age and then cleared immediately (hypothesis D1).

**Hypothesis D1** RBCs are cleared at rate 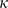 until age 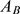, any remaining are then immediately cleared.

No modification to Equation 2 is required.

**Hypothesis D2** No clearance of RBCs until they reach age 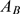, they are then rapidly cleared.

This means 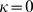 in Equation 2.

**Hypothesis D3** RBCs are cleared at rate 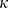.

This means 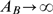 in Equation 2.

#### Production of reticulocyte spikes

Spikes in reticulocyte proportion have not previously been documented in the literature. This could be because sampling is rarely undertaken more frequently than once per day, so a spike lasting for less than 24 hours would not be observed. Or it could be because spikes have previously been interpreted as experimental noise.

Our control experiments strongly suggest that reticulocyte spikes are induced by blood sampling and not by any intrinsic response to anaemia. Moreover, we have found no biologically plausible mechanism that can produce reticulocyte spikes at non-periodic intervals. Therefore, we model them phenomenologically (that is, without any mechanistic explanation) as a series of pulsed inputs of reticulocytes either into bone marrow (hypothesis E2) or directly into the circulation (hypothesis E1). We also test if our base model can provide an adequate fit to our data without including reticulocyte spikes (hypothesis E3). This phenomenological model can be thought of as a filter, filtering out nuisance data in order to extract information from the underlying data. Nevertheless, these spikes do change RBC and reticulocyte dynamics, so we may be able to make inferences about their mechanistic cause.

**Hypothesis E1** Sampling-induced reticulocytes enter directly into the circulation. Their production rate and maturation time may be different to normal reticulocytes.

Equation 2 is modified by the additional term
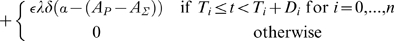
(21)where 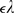 is the production rate of sampling-induced reticulocytes, 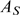 is their maturation time in the the circulation, 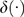 is the Dirac delta function, 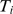 is the time after PHZ administration that spike 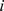 appears, 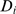 is the duration of spike 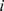 and 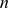 the number of spikes.

**Hypothesis E2** Sampling-induced reticulocytes are produced in bone marrow. Their maturation time is the same as normal reticulocytes but their production rate may be different.

Equation 3 is modified by the additional term
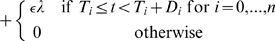
(22)


**Hypothesis E3** No sampling-induced reticulocytes.

No modifications to Equations 1 and 3 are required.

### Statistical analysis

#### Measurement error in RBC concentration

Close to the end of the main experiment we used seven of the mice to determine the error structure and variance of the RBC concentration measurements. For each mouse, four 2 µl blood samples were taken and coulter counted. If the measurement error variance is 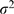, then the difference between any pair of samples from a mouse has expectation 0 and variance 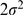. For each mouse there are two independent pairs, and therefore, with 7 mice, 14 independent pairs in total. The errors were found to be normally distributed (Anderson-Darling test 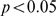 [47]) and the estimate of 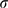 was 0.61×10^9^ (95% CI: 0.44×10^9^, 0.84×10^9^) cells/ml.

We further refined our estimate of 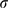 using the main experiment data during the model fitting. Our prior on 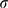 was taken as normally distributed with mean 0.61×10^9^ and standard deviation 0.1×10^9^ (based on the estimated 95% CI of 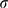).

#### Measurement error in reticulocyte counts

For each smear, the number of reticulocytes and normocytes were counted from a random sample of 500 RBCs (except at the first time point for mice 1 and 2 when 2000 RBCs were counted, and at the first time point for the other mice when 1000 RBCs were counted). If the true proportion of reticulocytes in mouse 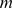 at time 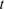 is 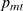 then the number of counted reticulocytes 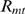, is Binomially distributed with parameters 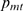 and 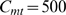, 1000 or 2000.

#### Model fitting and parameter estimation

The models were numerically implemented in discrete time with a timestep of 1 hr. We used an Evolutionary Monte Carlo (EMC) method [48] to sample the posterior density. Parameters were estimated separately for each mouse, except the RBC concentration measurement error 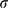 which was simultaneously estimated across all mice. We did not estimate any hyper parameters.

The parameter 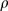 was not directly estimated, but was derived by solving the following equation for 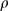 using the bisection method with tolerance 10^−5^

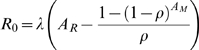
(23)where 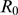 is the initial circulating reticulocyte concentration which was also estimated. This equation was derived by considering the total concentration of reticulocytes in bone marrow and circulation (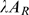) and subtracting from that the concentration of reticulocytes in bone marrow. The equation is approximate because we have not included random clearance of reticulocytes, but this effect will be small.

The likelihood of a model with parameter vector 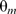 given the data for mouse 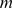 is, up to a constant of proportionality,
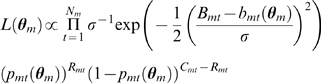
(24)where 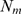 is the number of data points for mouse 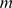, 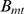 is the coulter counter measured RBC concentration at time point 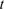, 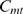 is the number of RBCs counted on a smear (500, 1000 or 2000), 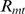 is the number of reticulocytes counted, 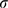 is the RBC measurement error, 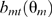 is the model solution of RBC concentration at time 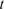, 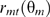 is the model solution of reticulocyte concentration at time 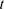, and 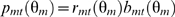 is the model solution of reticulocyte proportion at time 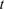. Most of the priors on the parameters were uniformly distributed, their ranges roughly based on pre-model fitting estimates. Based on previous estimates, the prior on the maximum RBC lifespan 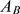 was 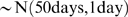 [33] which helps identifiability of reticulocyte production rate 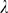 and RBC clearance rate 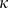.

The Markov chain Monte Carlo jumping distributions were multivariate normal with covariance matrices estimated from a empirical covariance matrices calculated during an adaptive phase [49]. The scale of the covariance matrices were tuned to give an acceptance rate between 20 and 40% [50]. Our inferences are based on 10^4^ samples, thinned from 2×10^6^ iterations of a non-adaptive Markov chain with the first half of the chain discarded as burn-in.

#### Model adequacy and comparison

We assess model adequacy with standardised residual plots. By overlaying plots for each mouse on one graph, the power of the plots to reveal poor fit is improved. We also plot the fits to the data with 95% posterior predictive intervals. These intervals predict where 95% of data would lie given the model being true and the posterior distribution. A poor fitting model would have significantly less than 95% of the data lying within this interval. These intervals are constructed by simulating the model using the parameter vectors from the Markov chain and generating replicate data sets with the known error structures and variances. At a given time point the 95% interval is taken to lie between the 2.5 and 97.5 percentiles of the distribution of replicated data.

To compare models we use Bayes factors [51]. We compare all models to the model consisting of hypotheses A1, B1, C1, D1 and E1. To calculate Bayes factors we need to calculate the marginal likelihood of a model, 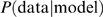 [52]. The Bayes factor of model 1 and model 2 is
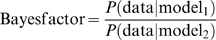
(25)It is more convenient to work in decibans (tenths of a power of 10)
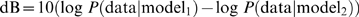
(26)A dB<5 suggests almost no difference between the models, a dB between 5 and 10 suggest substantial evidence in favour of model 1 and a dB>10 is strong evidence in favour of model 1 [51].
